# The genome sequence of the Cockspur worm, *Bimastos rubidus* (Savigny, 1826) (Crassiclitellata: Lumbricidae)

**DOI:** 10.12688/wellcomeopenres.24961.1

**Published:** 2025-10-10

**Authors:** Emma Sherlock, Keiron D. Brown, Chris Fletcher, Natural History Museum Genome Acquisition Lab, Darwin Tree of Life Barcoding Collective, Wellcome Sanger Institute Tree of Life Management, Samples and Laboratory team, Wellcome Sanger Institute Scientific Operations: Sequencing Operations, Wellcome Sanger Institute Tree of Life Core Informatics team, Tree of Life Core Informatics collective, Darwin Tree of Life Consortium

**Affiliations:** 1 Natural History Museum, London, England, UK; 2 Biological Recording Company, Uxbridge, England, UK

**Keywords:** Bimastos rubidus; Cockspur worm; genome sequence; chromosomal; Crassiclitellata

## Abstract

We present a genome assembly from an individual *Bimastos rubidus* (Cockspur worm; Annelida; Clitellata; Crassiclitellata; Lumbricidae). The genome sequence has a total length of 812.74 megabases. Most of the assembly (95.63%) is scaffolded into 17 chromosomal pseudomolecules. The mitochondrial genome has also been assembled, with a length of 16.57 kilobases. This assembly was generated as part of the Darwin Tree of Life project, which produces reference genomes for eukaryotic species found in Britain and Ireland.

## Species taxonomy

Eukaryota; Opisthokonta; Metazoa; Eumetazoa; Bilateria; Protostomia; Spiralia; Lophotrochozoa; Annelida; Clitellata; Oligochaeta; Crassiclitellata; Lumbricina; Lumbricidae; Lumbricinae; Bimastos; *Bimastos rubidus* (Savigny, 1826) (NCBI:txid2866284)

## Background

*Bimastos rubidus* (European bark worm, bank worm, or cockspur worm) is identified by its external morphology, including an epilobic head; a clitellum starting on segments 26 or 27 and extending to 31, 32, or 33; and a tubercula pubertatis (TP) beginning on segments 28 or 29 and ending on 30 or 31. The TP is a swelling that often appears as a band or block. Internal distinguishing features include three pairs of seminal vesicles in segments 9, 11, and 12, and two pairs of spermathecae in intersegments 9/10 and 10/11 ( Sherlock, 2018).

In the field, this worm is noted for its deep red dorsal colouration. It is typically found under bark of rotting wood, beneath stones or dung, in soils rich in organic matter, or in association with wood ant nests. It belongs to the ecological group epigeic and the functional group litter dweller. It occurs in a wide range of habitats but shows a preference for woodland, and is only occasionally recorded from farmland ( Ashwood *et al.*, 2024).

*Bimastos rubidus* was formerly classified in the genus *Dendrodrilus*, but has been reassigned to *Bimastos* (still within Lumbricidae) based on recent molecular phylogenetic studies ( Csuzdi *et al.*, 2017).

**Figure 1.**
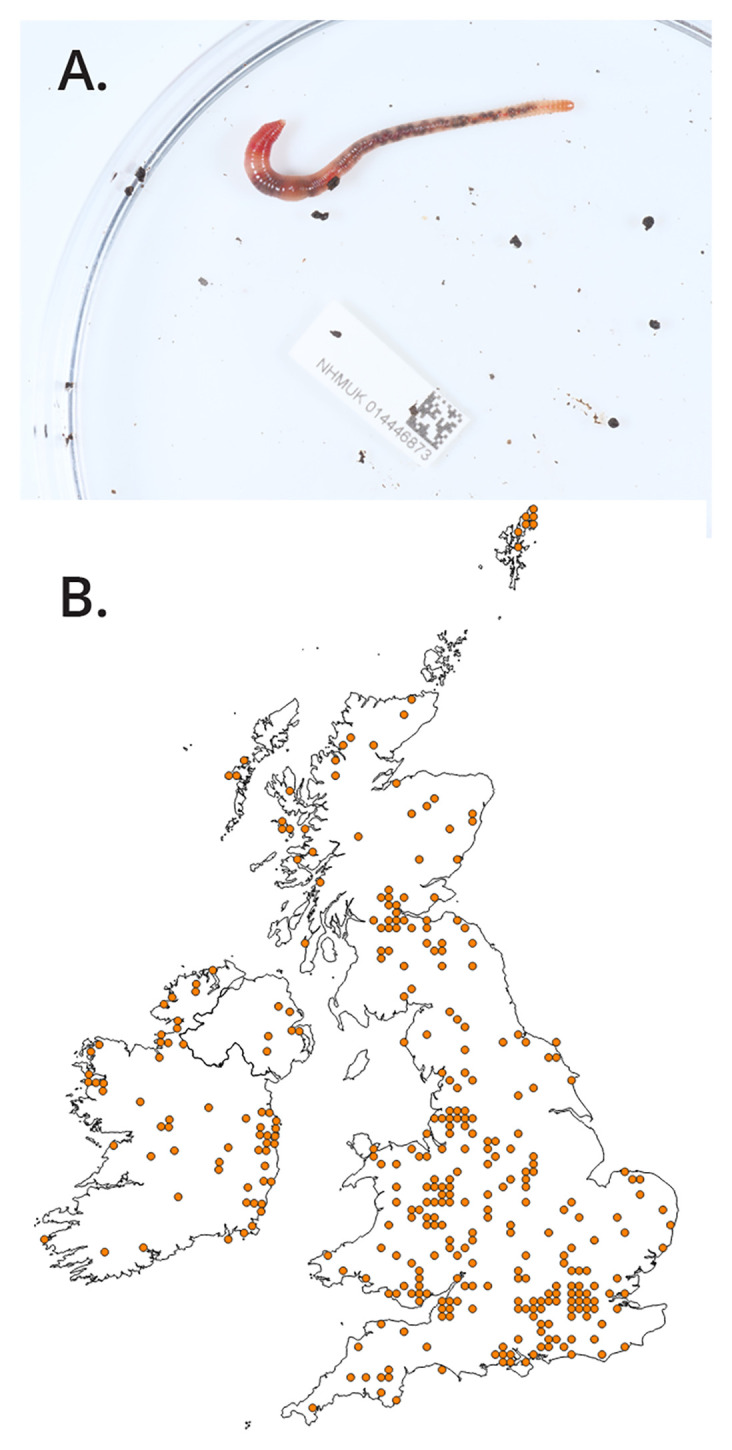
**A**. Photograph of the *Bimastos rubidus* (whBimRubi4) specimen used for genome sequencing. **B**. Distribution map of *B. rubidus*, generated from the National Earthworm Recording Scheme dataset 2025 (created by Rich Burkmar).

This species was until recently considered native to Europe, but is now thought to be of North American origin ( Csuzdi *et al.*, 2017). It is known from Europe, North America, South America, Asia, Africa, and Australasia. It occurs in all four countries of the United Kingdom as well as the Republic of Ireland. The widespread nature of records across the UK is shown in Figure 1 B.

We present a chromosome-level genome sequence for *Bimastos rubidus*, generated by the Tree of Life pipeline from a specimen collected in Sydenham Hill Woods, England, UK ( Figure 1). This assembly was produced as part of the Darwin Tree of Life Project, which aims to generate high-quality reference genomes for all named eukaryotic species in Britain and Ireland to support research, conservation, and sustainable use of biodiversity ( Blaxter *et al.*, 2022).

## Methods

### Sample acquisition and DNA barcoding

The specimen used for genome sequencing was an adult *Bimastos rubidus* (specimen ID NHMUK014446873, ToLID whBimRubi4; Figure 1), collected from Sydenham Hill Woods, England, United Kingdom (latitude 51.435, longitude –0.0735) on 2020-10-07. A second specimen used for Hi-C sequencing (specimen ID NHMUK014446993, ToLID whBimRubi3) was collected on the same occasion. Emma Sherlock and Chris Fletcher collected the specimens and Emma Sherlock formally identified them. A third specimen (specimen ID Ox001366, ToLID whBimRubi1) used for RNA sequencing was collected from Wytham Woods, Oxfordshire, United Kingdom (latitude 51.776, longitude –1.341) on 2021-05-27. This specimen was collected and identified by Keiron Brown. Sample metadata were collected in line with the Darwin Tree of Life project standards described by Lawniczak *et al*. (2022).

The initial identification was verified by an additional DNA barcoding process according to the framework developed by Twyford *et al.* (2024). A small sample was dissected from the specimen and stored in ethanol, while the remaining parts were shipped on dry ice to the Wellcome Sanger Institute (WSI) (see the protocol). The tissue was lysed, the COI marker region was amplified by PCR, and amplicons were sequenced and compared to the BOLD database, confirming the species identification ( Crowley *et al.*, 2023). Following whole genome sequence generation, the relevant DNA barcode region was also used alongside the initial barcoding data for sample tracking at the WSI ( Twyford *et al.*, 2024). The standard operating procedures for Darwin Tree of Life barcoding are available on protocols.io.

### Nucleic acid extraction

Protocols for high molecular weight (HMW) DNA extraction developed at the Wellcome Sanger Institute (WSI) Tree of Life Core Laboratory are available on protocols.io ( Howard *et al.*, 2025). The whBimRubi4 sample was weighed and triaged to determine the appropriate extraction protocol. Tissue from the posterior body was homogenised by powermashing using a PowerMasher II tissue disruptor. HMW DNA was extracted using the Automated MagAttract v2 protocol. DNA was sheared into an average fragment size of 12–20 kb following the Megaruptor®3 for LI PacBio protocol. Sheared DNA was purified by automated SPRI (solid-phase reversible immobilisation). The concentration of the sheared and purified DNA was assessed using a Nanodrop spectrophotometer and Qubit Fluorometer using the Qubit dsDNA High Sensitivity Assay kit. Fragment size distribution was evaluated by running the sample on the FemtoPulse system. For this sample, the final post-shearing DNA had a Qubit concentration of 20.2 ng/μL and a yield of 2 626.00 ng.

RNA was extracted from posterior body tissue of whBimRubi1 in the Tree of Life Laboratory at the WSI using the RNA Extraction: Automated MagMax™ *mir*Vana protocol. The RNA concentration was assessed using a Nanodrop spectrophotometer and a Qubit Fluorometer using the Qubit RNA Broad-Range Assay kit. Analysis of the integrity of the RNA was done using the Agilent RNA 6000 Pico Kit and Eukaryotic Total RNA assay.

### PacBio HiFi library preparation and sequencing

Library preparation and sequencing were performed at the WSI Scientific Operations core. Libraries were prepared using the SMRTbell Prep Kit 3.0 (Pacific Biosciences, California, USA), following the manufacturer’s instructions. The kit includes reagents for end repair/A-tailing, adapter ligation, post-ligation SMRTbell bead clean-up, and nuclease treatment. Size selection and clean-up were performed using diluted AMPure PB beads (Pacific Biosciences). DNA concentration was quantified using a Qubit Fluorometer v4.0 (ThermoFisher Scientific) and the Qubit 1X dsDNA HS assay kit. Final library fragment size was assessed with the Agilent Femto Pulse Automated Pulsed Field CE Instrument (Agilent Technologies) using the gDNA 55 kb BAC analysis kit.

The sample was sequenced on a Revio instrument (Pacific Biosciences). The prepared library was normalised to 2 nM, and 15 μL was used for making complexes. Primers were annealed and polymerases bound to generate circularised complexes, following the manufacturer’s instructions. Complexes were purified using 1.2X SMRTbell beads, then diluted to the Revio loading concentration (200–300 pM) and spiked with a Revio sequencing internal control. The sample was sequenced on a Revio 25M SMRT cell. The SMRT Link software (Pacific Biosciences), a web-based workflow manager, was used to configure and monitor the run and to carry out primary and secondary data analysis.

### Hi-C

***Sample preparation and crosslinking***

The Hi-C sample was prepared from 20–50 mg of frozen tissue from the posterior body of the whBimRubi3 sample using the Arima-HiC v2 kit (Arima Genomics). Following the manufacturer’s instructions, tissue was fixed and DNA crosslinked using TC buffer to a final formaldehyde concentration of 2%. The tissue was homogenised using the Diagnocine Power Masher-II. Crosslinked DNA was digested with a restriction enzyme master mix, biotinylated, and ligated. Clean-up was performed with SPRISelect beads before library preparation. DNA concentration was measured with the Qubit Fluorometer (Thermo Fisher Scientific) and Qubit HS Assay Kit. The biotinylation percentage was estimated using the Arima-HiC v2 QC beads.

***Hi-C library preparation and sequencing***

Biotinylated DNA constructs were fragmented using a Covaris E220 sonicator and size selected to 400–600 bp using SPRISelect beads. DNA was enriched with Arima-HiC v2 kit Enrichment beads. End repair, A-tailing, and adapter ligation were carried out with the NEBNext Ultra II DNA Library Prep Kit (New England Biolabs), following a modified protocol where library preparation occurs while DNA remains bound to the Enrichment beads. Library amplification was performed using KAPA HiFi HotStart mix and a custom Unique Dual Index (UDI) barcode set (Integrated DNA Technologies). Depending on sample concentration and biotinylation percentage determined at the crosslinking stage, libraries were amplified with 10–16 PCR cycles. Post-PCR clean-up was performed with SPRISelect beads. Libraries were quantified using the AccuClear Ultra High Sensitivity dsDNA Standards Assay Kit (Biotium) and a FLUOstar Omega plate reader (BMG Labtech).

Prior to sequencing, libraries were normalised to 10 ng/μL. Normalised libraries were quantified again and equimolar and/or weighted 2.8 nM pools. Pool concentrations were checked using the Agilent 4200 TapeStation (Agilent) with High Sensitivity D500 reagents before sequencing. Sequencing was performed using paired-end 150 bp reads on the Illumina NovaSeq 6000.

### RNA library preparation and sequencing

Libraries were prepared using the NEBNext ^®^ Ultra™ II Directional RNA Library Prep Kit for Illumina (New England Biolabs), following the manufacturer’s instructions. Poly(A) mRNA in the total RNA solution was isolated using oligo(dT) beads, converted to cDNA, and uniquely indexed; 14 PCR cycles were performed. Libraries were size-selected to produce fragments between 100–300 bp. Libraries were quantified, normalised, pooled to a final concentration of 2.8 nM, and diluted to 150 pM for loading. Sequencing was carried out on the Illumina NovaSeq 6000 to generate 150-bp paired-end reads.

### Genome assembly

Prior to assembly of the PacBio HiFi reads, a database of *k*-mer counts ( *k* = 31) was generated from the filtered reads using FastK. GenomeScope2 ( Ranallo-Benavidez *et al.*, 2020) was used to analyse the *k*-mer frequency distributions, providing estimates of genome size, heterozygosity, and repeat content.

The HiFi reads were assembled using Hifiasm ( Cheng *et al.*, 2021) with the --primary option. The Hi-C reads ( Rao *et al.*, 2014) were mapped to the primary contigs using bwa-mem2 ( Vasimuddin *et al.*, 2019), and the contigs were scaffolded in YaHS ( Zhou *et al.*, 2023) with the --break option for handling potential misassemblies. The scaffolded assemblies were evaluated using Gfastats ( Formenti *et al.*, 2022), BUSCO ( Manni *et al.*, 2021) and MERQURY.FK ( Rhie *et al.*, 2020).

The mitochondrial genome was assembled using MitoHiFi ( Uliano-Silva *et al.*, 2023), which runs MitoFinder ( Allio *et al.*, 2020) and uses these annotations to select the final mitochondrial contig and to ensure the general quality of the sequence.

### Assembly curation

The assembly was decontaminated using the Assembly Screen for Cobionts and Contaminants ( ASCC) pipeline. TreeVal was used to generate the flat files and maps for use in curation. Manual curation was conducted primarily in PretextView and HiGlass ( Kerpedjiev *et al.*, 2018). Scaffolds were visually inspected and corrected as described by Howe *et al*. (2021). Manual corrections included 171 breaks, 153 joins, and removal of 83 haplotypic duplications. The curation process is documented at https://gitlab.com/wtsi-grit/rapid-curation. PretextSnapshot was used to generate a Hi-C contact map of the final assembly.

### Assembly quality assessment

The Merqury.FK tool ( Rhie *et al.*, 2020) was run in a Singularity container ( Kurtzer *et al.*, 2017) to evaluate *k*-mer completeness and assembly quality for the primary and alternate haplotypes using the *k*-mer databases ( *k* = 31) computed prior to genome assembly. The analysis outputs included assembly QV scores and completeness statistics.

The genome was analysed using the BlobToolKit pipeline, a Nextflow implementation of the earlier Snakemake version ( Challis *et al.*, 2020). The pipeline aligns PacBio reads using minimap2 ( Li, 2018) and SAMtools ( Danecek *et al.*, 2021) to generate coverage tracks. It runs BUSCO ( Manni *et al.*, 2021) using lineages identified from the NCBI Taxonomy ( Schoch *et al.*, 2020). For the three domain-level lineages, BUSCO genes are aligned to the UniProt Reference Proteomes database ( Bateman *et al.*, 2023) using DIAMOND blastp ( Buchfink *et al.*, 2021). The genome is divided into chunks based on the density of BUSCO genes from the closest taxonomic lineage, and each chunk is aligned to the UniProt Reference Proteomes database with DIAMOND blastx. Sequences without hits are chunked using seqtk and aligned to the NT database with blastn ( Altschul *et al.*, 1990). The BlobToolKit suite consolidates all outputs into a blobdir for visualisation. The BlobToolKit pipeline was developed using nf-core tooling ( Ewels *et al.*, 2020) and MultiQC ( Ewels *et al.*, 2016), with containerisation through Docker ( Merkel, 2014) and Singularity ( Kurtzer *et al.*, 2017).

## Genome sequence report

### Sequence data

**Figure 2.**
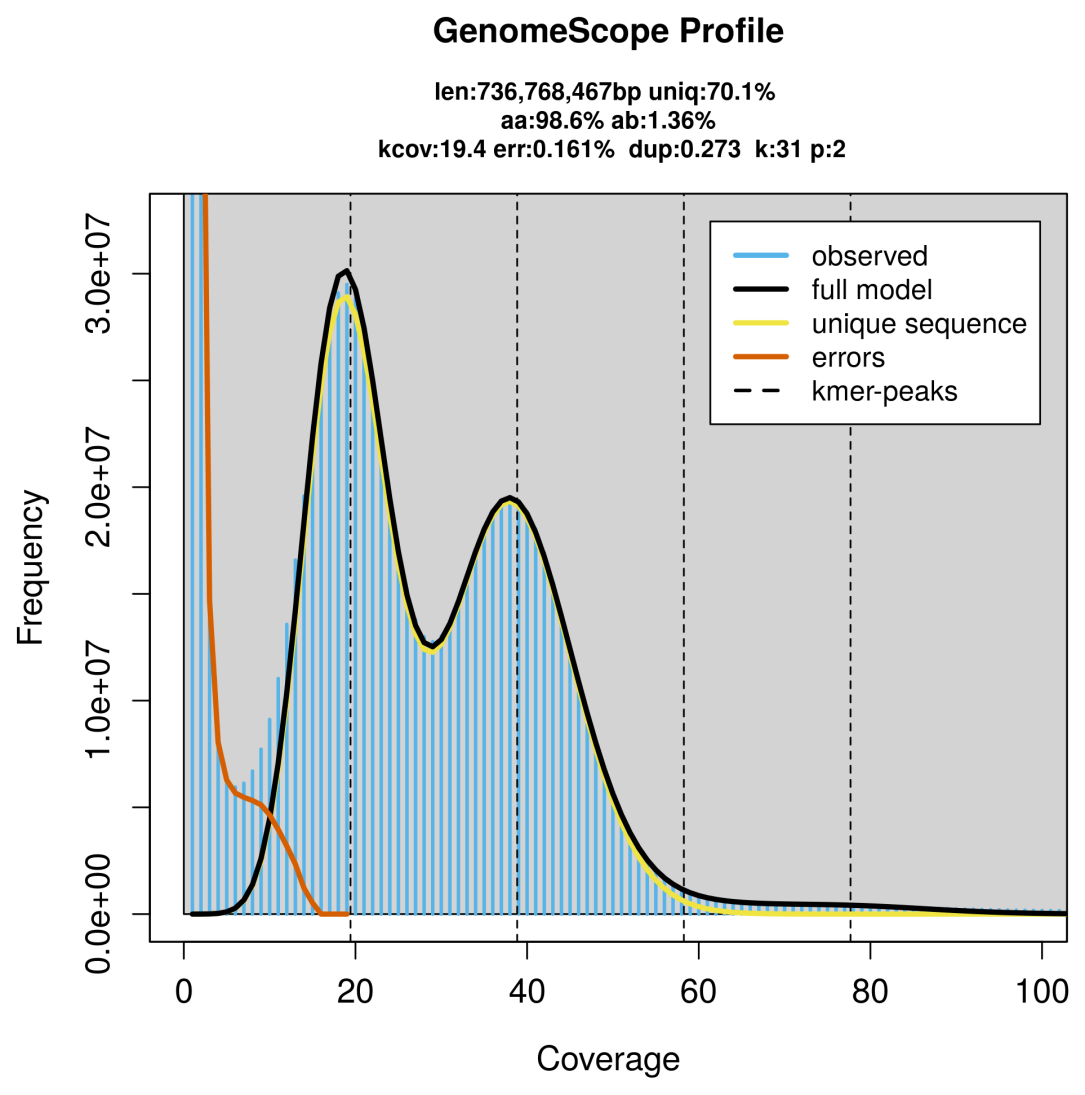
Frequency distribution of *k*-mers generated using GenomeScope2. The plot shows observed and modelled *k*-mer spectra, providing estimates of genome size, heterozygosity, and repeat content based on unassembled sequencing reads.

PacBio sequencing of the *Bimastos rubidus* specimen generated 30.20 Gb (gigabases) from 3.56 million reads, which were used to assemble the genome. GenomeScope2.0 analysis estimated the haploid genome size at 736.77 Mb, with a heterozygosity of 1.36% and repeat content of 30.02% ( Figure 2). These estimates guided expectations for the assembly. Based on the estimated genome size, the sequencing data provided approximately 39× coverage. Hi-C sequencing produced 114.49 Gb from 758.19 million reads, which were used to scaffold the assembly. RNA sequencing data were also generated and are available in public sequence repositories. Table 1 summarises the specimen and sequencing details.

**Table 1.** Specimen and sequencing data for BioProject PRJEB76847.

Platform	PacBio HiFi	Hi-C	RNA-seq
**ToLID**	whBimRubi4	whBimRubi3	whBimRubi1
**Specimen ID**	NHMUK014446873	NHMUK014446993	Ox001366
**BioSample (source individual)**	SAMEA10241715	SAMEA10241714	SAMEA10166838
**BioSample (tissue)**	SAMEA10241741	SAMEA10241735	SAMEA10201131
**Tissue**	posterior body	posterior body	posterior body
**Instrument**	Revio	Illumina NovaSeq 6000	Illumina NovaSeq 6000
**Run accessions**	ERR13304165	ERR13317844	ERR13317845
**Read count total**	3.56 million	758.19 million	58.56 million
**Base count total**	30.20 Gb	114.49 Gb	8.84 Gb

### Assembly statistics

The primary haplotype was assembled, and contigs corresponding to an alternate haplotype were also deposited in INSDC databases. The final assembly has a total length of 812.74 Mb in 414 scaffolds, with 1 740 gaps, and a scaffold N50 of 49.71 Mb ( Table 2).

**Table 2.** Genome assembly statistics.

**Assembly name**	whBimRubi4.1
**Assembly accession**	GCA_964330305.1
**Alternate haplotype accession**	GCA_964330325.1
**Assembly level**	chromosome
**Span (Mb)**	812.74
**Number of chromosomes**	17
**Number of contigs**	2 154
**Contig N50**	0.77 Mb
**Number of scaffolds**	414
**Scaffold N50**	49.71 Mb
**Sex chromosomes**	N/A
**Organelles**	Mitochondrion: 16.57 kb

**Figure 3.**
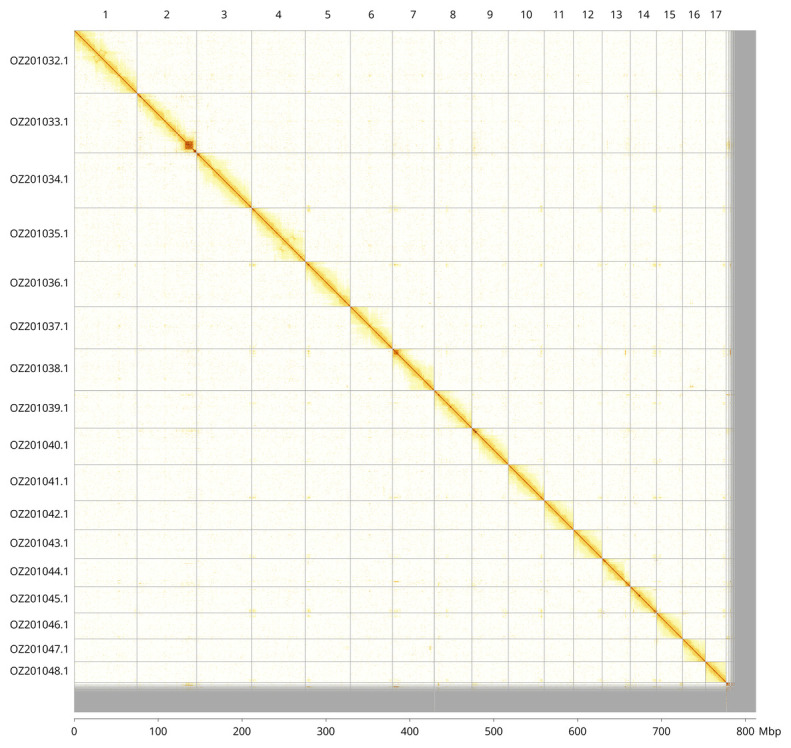
Hi-C contact map of the *Bimastos rubidus* genome assembly. Assembled chromosomes are shown in order of size and labelled along the axes, with a megabase scale shown below. The plot was generated using PretextSnapshot.

Most of the assembly sequence (95.63%) was assigned to 17 chromosomal-level scaffolds. These chromosome-level scaffolds, confirmed by Hi-C data, are named according to size ( Figure 3; Table 3).

**Table 3.** Chromosomal pseudomolecules in the primary genome assembly of *Bimastos rubidus* whBimRubi4.

INSDC accession	Molecule	Length (Mb)	GC%
OZ201032.1	1	74.99	40
OZ201033.1	2	70.97	39.50
OZ201034.1	3	65.62	40
OZ201035.1	4	64.05	40
OZ201036.1	5	53.49	40
OZ201037.1	6	50.26	40.50
OZ201038.1	7	49.85	40
OZ201039.1	8	44.70	40.50
OZ201040.1	9	43.64	40.50
OZ201041.1	10	42.76	40.50
OZ201042.1	11	34.89	40.50
OZ201043.1	12	34.25	40.50
OZ201044.1	13	33.41	40
OZ201045.1	14	31.44	40.50
OZ201046.1	15	31	40.50
OZ201047.1	16	27.07	40.50
OZ201048.1	17	24.81	41

The mitochondrial genome was also assembled. This sequence is included as a contig in the multifasta file of the genome submission and as a standalone record.

**Figure 4.**
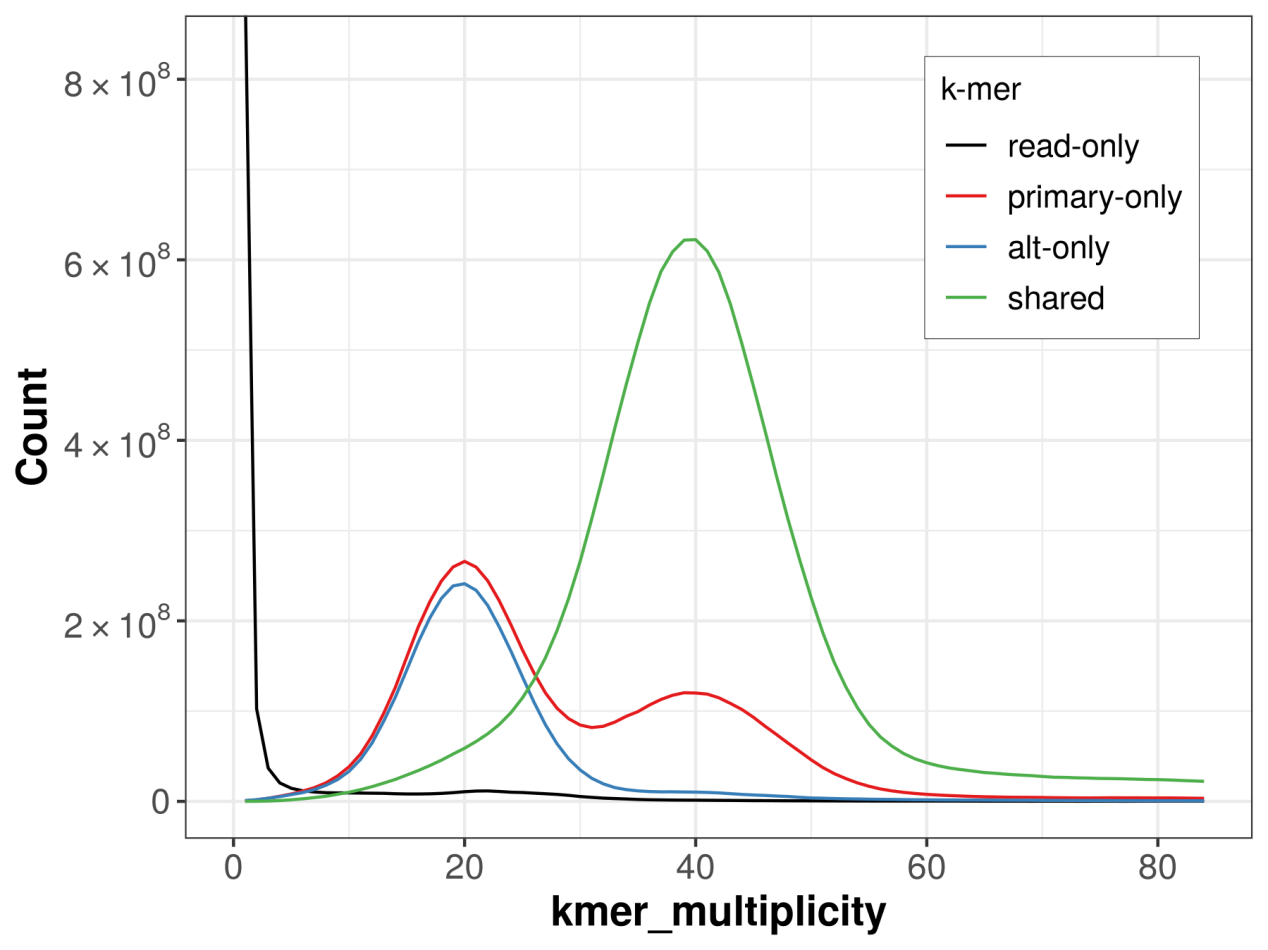
Evaluation of *k*-mer completeness using MerquryFK. This plot illustrates the recovery of *k*‐mers from the original read data in the final assemblies. The horizontal axis represents *k*‐mer multiplicity, and the vertical axis shows the number of *k*‐mers. The black curve represents *k*‐mers that appear in the reads but are not assembled. The green curve corresponds to *k*‐mers shared by both haplotypes, and the red and blue curves show *k*‐mers found only in one of the haplotypes.

The combined primary and alternate assemblies achieve an estimated QV of 54.9. The *k*-mer completeness is 76.62% for the primary assembly, 67.42% for the alternate haplotype, and 97.99% for the combined assemblies ( Figure 4).

**Figure 5.**
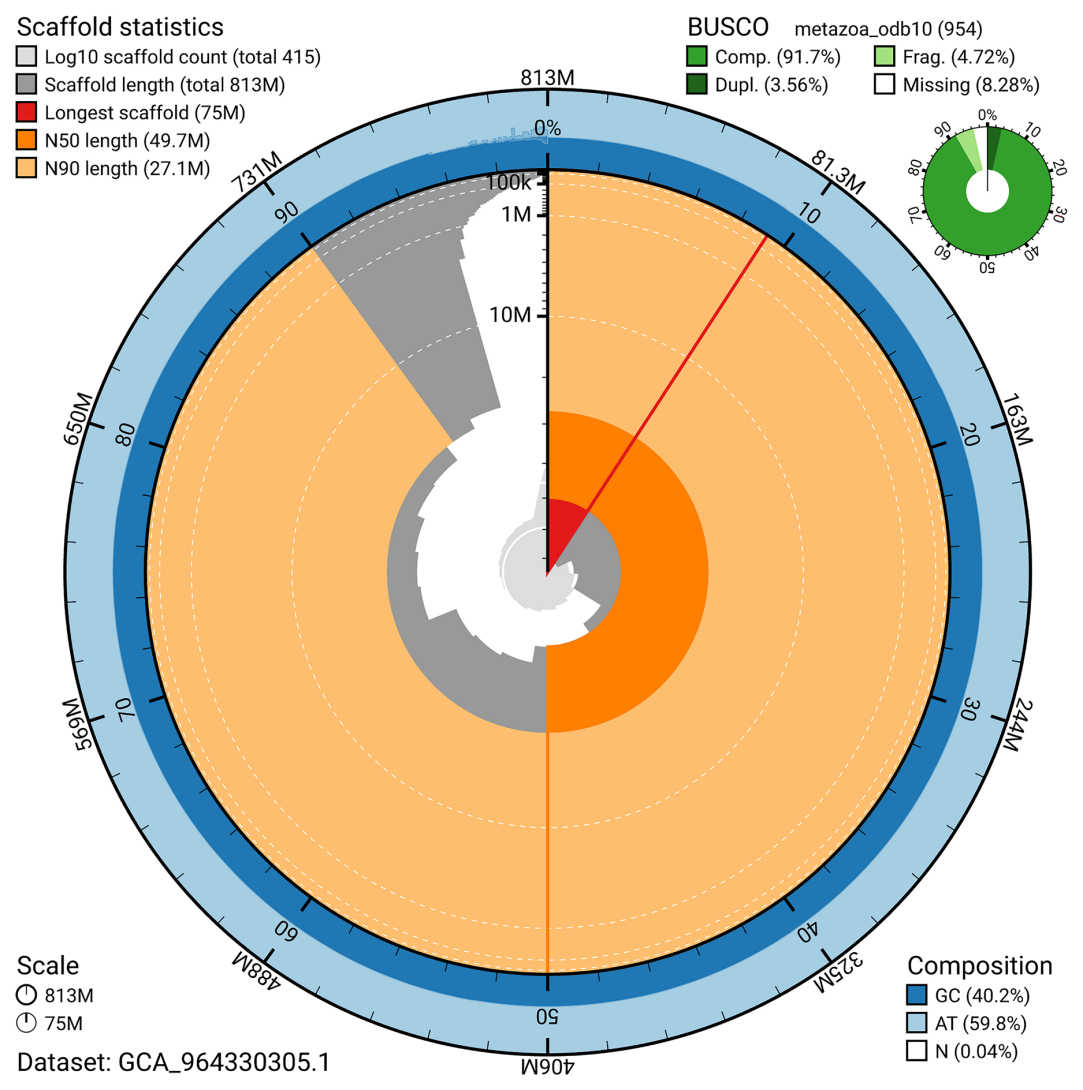
Assembly metrics for whBimRubi4.1. The BlobToolKit snail plot provides an overview of assembly metrics and BUSCO gene completeness. The circumference represents the length of the whole genome sequence, and the main plot is divided into 1 000 bins around the circumference. The outermost blue tracks display the distribution of GC, AT, and N percentages across the bins. Scaffolds are arranged clockwise from longest to shortest and are depicted in dark grey. The longest scaffold is indicated by the red arc, and the deeper orange and pale orange arcs represent the N50 and N90 lengths. A light grey spiral at the centre shows the cumulative scaffold count on a logarithmic scale. A summary of complete, fragmented, duplicated, and missing BUSCO genes in the metazoa_odb10 set is presented at the top right. An interactive version of this figure can be accessed on the BlobToolKit viewer.

**Figure 6.**
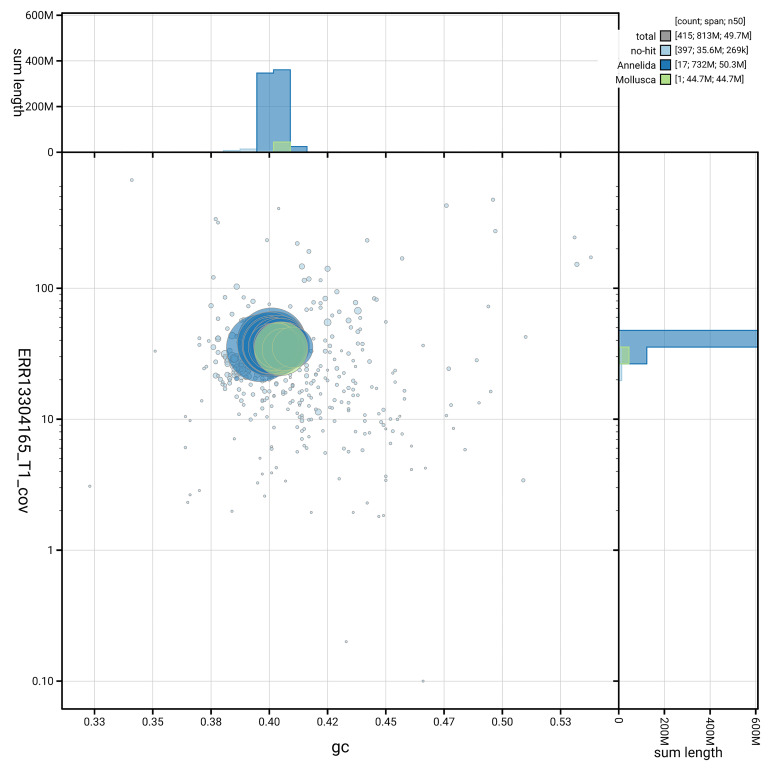
BlobToolKit GC-coverage plot for whBimRubi4.1. Blob plot showing sequence coverage (vertical axis) and GC content (horizontal axis). The circles represent scaffolds, with the size proportional to scaffold length and the colour representing phylum membership. The histograms along the axes display the total length of sequences distributed across different levels of coverage and GC content. An interactive version of this figure is available on the BlobToolKit viewer.

BUSCO v.5.5.0 analysis using the metazoa_odb10 reference set ( *n* = 954) identified 91.7% of the expected gene set (single = 88.2%, duplicated = 3.6%). The snail plot in Figure 5 summarises the scaffold length distribution and other assembly statistics for the primary assembly. The blob plot in Figure 6 shows the distribution of scaffolds by GC proportion and coverage.

Table 4 lists the assembly metric benchmarks adapted from Rhie *et al.* (2021) the Earth BioGenome Project Report on Assembly Standards September 2024. The EBP metric, calculated for the primary assembly, is **5.C.Q54**.

**Table 4.** Earth Biogenome Project summary metrics for the *Bimastos rubidus* assembly.

Measure	Value	Benchmark
EBP summary (primary)	5.C.Q54	6.C.Q40
Contig N50 length	0.77 Mb	≥ 1 Mb
Scaffold N50 length	49.71 Mb	= chromosome N50
Consensus quality (QV)	Primary: 54.6; alternate: 55.2; combined: 54.9	≥ 40
*k*-mer completeness	Primary: 76.62%; alternate: 67.42%; combined: 97.99%	≥ 95%
BUSCO	C:91.7% [S:88.2%; D:3.6%]; F:4.7%; M:3.6%; n:954	S > 90%; D < 5%
Percentage of assembly assigned to chromosomes	95.63%	≥ 90%

### Wellcome Sanger Institute – Legal and Governance

The materials that have contributed to this genome note have been supplied by a Darwin Tree of Life Partner. The submission of materials by a Darwin Tree of Life Partner is subject to the **‘Darwin Tree of Life Project Sampling Code of Practice’**, which can be found in full on the Darwin Tree of Life website. By agreeing with and signing up to the Sampling Code of Practice, the Darwin Tree of Life Partner agrees they will meet the legal and ethical requirements and standards set out within this document in respect of all samples acquired for, and supplied to, the Darwin Tree of Life Project. Further, the Wellcome Sanger Institute employs a process whereby due diligence is carried out proportionate to the nature of the materials themselves, and the circumstances under which they have been/are to be collected and provided for use. The purpose of this is to address and mitigate any potential legal and/or ethical implications of receipt and use of the materials as part of the research project, and to ensure that in doing so we align with best practice wherever possible. The overarching areas of consideration are:

- Ethical review of provenance and sourcing of the material
- Legality of collection, transfer and use (national and international)

Each transfer of samples is further undertaken according to a Research Collaboration Agreement or Material Transfer Agreement entered into by the Darwin Tree of Life Partner, Genome Research Limited (operating as the Wellcome Sanger Institute), and in some circumstances, other Darwin Tree of Life collaborators.

## Data Availability

European Nucleotide Archive: Bimastos rubidus (bank worm). Accession number PRJEB76847. The genome sequence is released openly for reuse. The *Bimastos rubidus* genome sequencing initiative is part of the Darwin Tree of Life Project (PRJEB40665) and the Sanger Institute Tree of Life Programme (PRJEB43745). All raw sequence data and the assembly have been deposited in INSDC databases. The genome will be annotated using available RNA-Seq data and presented through the Ensembl pipeline at the European Bioinformatics Institute. Raw data and assembly accession identifiers are reported in Table 1 and Table 2. Production code used in genome assembly at the WSI Tree of Life is available at https://github.com/sanger-tol. Table 5 lists software versions used in this study.

## Author information

Contributors are listed at the following links:

- Members of the University of Oxford and Wytham Woods Genome Acquisition Lab
- Members of the Natural History Museum Genome Acquisition Lab
- Members of the Darwin Tree of Life Barcoding collective
- Members of the Wellcome Sanger Institute Tree of Life Management, Samples and Laboratory team
- Members of Wellcome Sanger Institute Scientific Operations – Sequencing Operations
- Members of the Wellcome Sanger Institute Tree of Life Core Informatics team
- Members of the Tree of Life Core Informatics collective
- Members of the Darwin Tree of Life Consortium
