# Supplementary material for: The genome sequence of the Cockspur worm, Bimastos rubidus (Savigny, 1826) (Crassiclitellata: Lumbricidae)
Source: Wellcome Open Res. 2025 Oct 10;10:563. [Version 1] doi: 10.12688/wellcomeopenres.24961.1 (PMC12576315; doi:10.12688/wellcomeopenres.24961.1)
Supplement: Peer review history

# Reviewer report: Reviewer response for version 1

**DOI:** 10.21956/wellcomeopenres.27496.r136046

**Published:** 2025-10-30

**Authors:** Jun Kim

**Affiliations:** 1 Chungnam National University, Daejeon, Daejeon, South Korea

This manuscript is well written, and the Wellcome Sanger Institute Tree of Life teams consistently produce high-quality and reliable genome assemblies. I have provided several comments below, but overall, this manuscript and its associated genome resources will serve as an invaluable foundation for future genetic and genomic studies of this species. I truly appreciate the authors’ efforts.

 Below are my comments to (hopefully) improve this manuscript:

 Page 4

 "Scientific) and the Qubit 1X dsDNA HS assay kit. Final"

 → “X” should be replaced with the multiplication symbol (×).

 "were purified using 1.2X SMRTbell beads, then diluted to the"

 → “X” should be replaced with the multiplication symbol (×).

 Page 5

 "The assembly was decontaminated using the Assembly Screen"

 → It would be helpful to specify whether this decontamination step affects the initial primary and alternate genome assemblies.

 "generate coverage tracks. It runs BUSCO (Manni et al., 2021)"

 → Would it be possible to integrate Compleasm, instead of the original version of BUSCO, into the BlobToolKit pipeline? This is not directly related to this manuscript, but it would be highly valuable for future applications within the Darwin Tree of Life Project.

 "ated 30.20 Gb (gigabases) from 3.56 million reads, which were"

 → It would be useful to clarify whether one Revio run produced only 30 Gb, or whether this run contained multiplexed samples. The amount of HiFi data seems somewhat small, so I am curious whether this is (1) due to the biological characteristics of the sample, or (2) the result of multiplexing, which might raise concerns about potential cross-sample contamination.

 "chromosomal-level scaffolds. These chromosome-level scaf"

 → It would be beneficial to examine whether these scaffolds contain telomeric repeat sequences at their termini. The tool seqtk telo can provide a quick assessment. If the telomeric motif differs from the canonical TTAGGG repeat, it would also be valuable to note this from an evolutionary perspective. [1,2]

 (For reference, the two papers I cited are actually my own, but they were mentioned merely as examples—there’s no need to cite them. I only wanted to emphasize that if the telomeric motif happens to differ, it would be quite interesting, so I’d really appreciate it if you could make sure to report it. Of course, I’d be grateful if you read those papers, but that’s entirely optional.)

 "The k-mer completeness is 76.62% for the primary assembly, 67.42% for the alternate haplotype,"

 → This relatively low k-mer completeness might result from incomplete assembly of repetitive regions or other technical factors. While this is not a major concern—given the difficulty of obtaining high-quality DNA from small invertebrates—it would be helpful to include potential explanations or cautions for this observation.

 Page 8

 "the Earth BioGenome Project Report on"

 → Please modify to: “the Earth BioGenome Project (EBP) Report on” to introduce the abbreviation.

Are sufficient details of methods and materials provided to allow replication by others?

Yes

Is the rationale for creating the dataset(s) clearly described?

Yes

Are the datasets clearly presented in a useable and accessible format?

Yes

Are the protocols appropriate and is the work technically sound?

Yes

Reviewer Expertise:

Genetics and genomics

I confirm that I have read this submission and believe that I have an appropriate level of expertise to confirm that it is of an acceptable scientific standard.

## References

[1] : Telomeric repeat evolution in the phylum Nematoda revealed by high-quality genome assemblies and subtelomere structures. *Genome Research* .2023;33(11) : 10.1101/gr.278124.123 1947-1957 10.1101/gr.278124.123 PMC1076044937918961

[2] : Whole-genome sequencing of 13 Arctic plants and draft genomes of Oxyria digyna and Cochlearia groenlandica. *Scientific Data* .2024;11(1) : 10.1038/s41597-024-03569-6 10.1038/s41597-024-03569-6 39025921 PMC11258133

# Reviewer report: Reviewer response for version 1

**DOI:** 10.21956/wellcomeopenres.27496.r136042

**Published:** 2025-10-16

**Authors:** Daniel F Marchán

**Affiliations:** 1 Complutense University of Madrid, Madrid, Community of Madrid, Spain

The work is methodologically sound, clearly written and includes all the information that could be expected.

 It is a worthy contribution to the genomic resources of earthworm species.

 I would suggest adding a small comment about the estimated number of chromosomes and previous cariological estimates, if they exist in the literature. If not for the same species, maybe for phylogenetically related species.

Are sufficient details of methods and materials provided to allow replication by others?

Yes

Is the rationale for creating the dataset(s) clearly described?

Yes

Are the datasets clearly presented in a useable and accessible format?

Yes

Are the protocols appropriate and is the work technically sound?

Yes

Reviewer Expertise:

Earthworm systematics, evolution and molecular phylogenetics

I confirm that I have read this submission and believe that I have an appropriate level of expertise to confirm that it is of an acceptable scientific standard.
